# Attenuated reward activations associated with cannabis use in anxious/depressed individuals

**DOI:** 10.1038/s41398-020-0807-9

**Published:** 2020-06-15

**Authors:** Philip A. Spechler, Jennifer L. Stewart, Rayus Kuplicki, the Tulsa 1000 Investigators, Robin Aupperle, Jerzy Bodurka, Justin Feinstein, Sahib S. Khalsa, Rayus Kuplicki, Martin P. Paulus, Jonathan Savitz, Jennifer Stewart, Teresa A. Victor, Martin P. Paulus

**Affiliations:** 1 Laureate Institute for Brain Research, 6655S. Yale Ave, Tulsa, OK 74136 USA; 2 University of Tulsa, Tulsa, OK USA

**Keywords:** Human behaviour, Neuroscience

## Abstract

Individuals with mood/anxiety disorders may use cannabis for “self-medication,” i.e., to induce positive mood or attenuate aversive mood states. However, little neurobiological evidence supports such use. The goal of this investigation was to test the hypothesis that cannabis use attenuates striatal response to reward in those with mood/anxiety disorders. Reward-related processing was measured using a monetary incentive delay task under functional MRI. Individuals with any lifetime mood/anxiety disorder diagnoses and problematic cannabis use (“Mood/Anxiety+CB”; *n* = 41) were compared with a propensity score-matched group of similar subjects without cannabis use (“Mood/Anxiety−CB”; *n* = 41), and a cannabis-naïve healthy control group (*n* = 35). Activations during win- and loss-anticipations were extracted from bilateral nucleus accumbens, dorsal caudate, and dorsolateral putamen. Mixed models were estimated for each region separately for win- and loss-anticipations, with a test for the main effect of group, condition (e.g., high-win, low-win, neutral), and their interaction. A significant main effect of group for win- and loss-anticipation was observed for each striatal region. Specifically, the Mood/Anxiety+CB group exhibited the lowest striatal activations across condition levels relative to both the Mood/Anxiety-CB and healthy group. A significant group-by-condition interaction was only observed for the dorsolateral putamen and indicated divergent activation modulation as a function of win and loss-magnitude for Mood/Anxiety+CB subjects. Finally, individuals with heavier recent cannabis use showed greater attenuation of gain-related activation in all three striatal regions. There was no such relationship for other illicit drugs. These data support the hypothesis that cannabis use in individuals with mood/anxiety disorders is associated with attenuated brain processing of reward magnitude, which may contribute to persistent affective symptoms.

## Introduction

Many individuals with mood or anxiety disorders believe that cannabis use might be a viable treatment option to alleviate their symptoms^1^. Individuals with these disorders may “self-medicate” with cannabis to achieve both the positive reinforcing (euphoric) and negative reinforcing (anxiolytic) effects of the drug^2^. Prevalence estimates from the National Epidemiologic Survey on Alcohol and Related Conditions^3^ indicate that 10% of individuals with mood disorders^4^ and 7.6% of individuals with anxiety disorders^5^ report any past year cannabis use. For context, data from the 2018 National Survey on Drug Use and Health report that 13.3% of individuals aged 26 and older have used cannabis in the past year^6^. While more research is needed to study the therapeutic potential of cannabis and affiliated compounds^7,8^, the literature frequently indicates that individuals with mood/anxiety disorders who use cannabis are likely to experience low symptom improvement^9,10^ and poor quality of life^4,5^.

A recent meta-analysis demonstrates that 52% of medical cannabis patients reported use for anxiety and 35% reported use for depression^11^. Moreover, epidemiological studies on drug risk perceptions show that perceived risk for regular cannabis use is low for adults and adolescents^6,12^, some of whom are likely to become the next generation of medical cannabis patients. These trends contrast with the lack of trials supporting cannabis as a safe or effective treatment for mood or anxiety disorders^8^, reflecting a disconnect between scientific evidence and public opinion. This paper therefore set out to extend our knowledge on the relationship between cannabis use and the functional neurobiology of individuals with mood/anxiety disorders.

Animal and human studies indicate that the reinforcing properties of cannabis are partially due to interactions with dopaminergic reward pathways in the brain^13,14^ typically achieved during acute intoxication. However, much less is known about the long-term effects of cannabis on reward circuitry. Dysregulated reward processing is a key feature of internalizing disorders, and is characterized by a reduced ability to anticipate positive affect. The monetary incentive delay (MID) task^15^ is one approach to examine neural substrates underlying reward-related processing; this paradigm involves the potential to win or avoid losing monetary gains and is broadly divided into anticipation and outcome phases separated by a time-varied delay period to allow for phase-specific blood-oxygen level dependent (BOLD) signals. The anticipation period reliably evokes signals from the dopaminergic-enriched striatum, while the outcome phase evokes signals across disparate cortical and subcortical regions^16,17^.

While the MID task has probed substance use and internalizing disorders^18,19^ little is known about the neural substrates underlying reward-related processing in cannabis use paired with depression/anxiety. The MID anticipation period probes approach behaviors thought to be dysregulated in both substance-using and depressed individuals, as rewarding nondrug incentives are devalued in both populations^20^. Despite the high concordance between substance use and internalizing disorders, functional magnetic resonance imaging (fMRI) studies including substance-using samples generally exclude individuals with psychopathologies^18^. Nonetheless, studies indicate that cannabis use^21,22^ and mood disorders^23^ are independently characterized by an attenuation of reward anticipation in striatal regions. Although there is mixed evidence for the relationship between cannabis use and win-anticipation (i.e., *increased* striatal activation^24,25^), differences in findings are likely due to inconsistent task parameters and inconsistent/untested sample characteristics, including psychiatric symptoms. Few studies have used the MID task to examine adult anxiety, although developmental work suggests that pediatric anxiety is characterized by an anticipatory hypersensitivity to wins and losses^26,27^. More research is needed to understand if this anxious hypersensitivity persists into adulthood when paired with substance use.

The present study examined whether lower striatal BOLD signals during MID win/loss anticipation would be observed in individuals with mood/anxiety disorders and cannabis use, compared with (1) a propensity score-matched group of cannabis-naïve individuals with similar internalizing symptoms, alcohol/nicotine dependence, and other sociodemographic variables; and (2) healthy controls without a history of mood/anxiety disorders or cannabis use. Propensity score-matching is an approach used to rigorously account for confounding variables by identifying groups roughly equated on a set of matching variables^28^ and is useful in strengthening quasi-causal conclusions drawn from observational studies^29^.

We specifically focused on MID anticipation (versus outcome) given prior work with substance use/internalizing disorder samples that showed striatal impairments were particularly observed during anticipation^21–23^. In addition, we used an *a-priori* region of interest (ROI) approach focused on striatal regions reliably recruited by the MID task^17,19^. The striatum can be broadly separated into ventromedial (nucleus accumbens) and dorsolateral (caudate, putamen) regions, such that the ventromedial area is involved in initial hedonic over-evaluation of drug rewards and acquisition of addictive behaviors, while the dorsolateral areas are involved in habituation of sensorimotor behaviors governing the maintenance of substance use disorders^30,31^.

In light of previous studies identifying lower striatal functioning in internalizing disorders^23^ and cannabis use^21,22^, we hypothesized an additive effect of the two, whereby individuals with cannabis use and internalizing disorders would exhibit lower striatal BOLD signals than the other two groups during reward anticipation. Evidence against this hypothesis would support the role of cannabis in sensitizing reward related processing in these individuals. However, evidence in support of this hypothesis would contextualize cannabis use for mood/anxiety symptoms as affiliated with a neurobiological disadvantage (i.e., lower striatal activations), and brings into question its use as a benign alternative treatment for internalizing symptoms. As atypical striatal activations have been shown to predict poor depression treatment outcomes^32,33^, and fewer days of cannabis use abstinence^34^, individuals with mood/anxiety disorders should be cautious of the likelihood of cannabis use to maintain and/or exacerbate the course of their mental health problems.

## Methods and materials

### T1000 study

Participants were drawn from the first 500 individuals recruited for the Tulsa 1000 (T1000) project, a naturalistic longitudinal study of 1000 individuals aged 18–65. The T1000 sample consisted of 1000 individuals seeking treatment for psychiatric symptoms^35^, including roughly 500 participants with mood/anxiety disorders, 300 with substance use disorders, 100 with eating disorders, and 100 healthy controls. Participants were recruited from the Laureate Psychiatric Clinic and Hospital, other local behavioral and mental health providers, and through newspaper, flyer, online, radio, and other media advertisements in the Tulsa metropolitan area. Participants with lifetime substance use disorders were referred from two local alcohol and drug treatment centers and screened for eligibility. The T1000 study was approved by the Western Institutional Review Board and adhered to the Declaration of Helsinki. All participants provided written informed consent and confidentiality was ensured. Participants first orally consented to complete a telephone or in-person screening to assess preliminary study eligibility.

The larger goal of the T1000 study was to identify latent factors across a suite of biobehavioral assessments to characterize mental health problems. The first 500 participants to complete the baseline assessments comprised an exploration dataset, while the later 500 were set aside as a validation set. Models and hypotheses generated from the first 500 participants, which include the data reported here, will later be evaluated for reproducibility on the set-aside 500 following publication and study pre-registration. See Victor et al. for complete study protocol and goals^35^.

Participants completed a clinical interview wherein trained staff administered the MINI International Neuropsychiatric Interview (version 6.0 or 7.0)^36^ to measure lifetime psychopathology in accordance with Diagnostic and Statistical Manual of Mental Disorders, 4th Edition or 5th Edition (DSM-5; American Psychiatric Association, 2013). Exclusion criteria for all groups were: (1) positive urine screen for alcohol/illicit drugs at clinical interview/neuroimaging sessions; (2) bipolar, obsessive compulsive, or schizophrenia spectrum disorders; (3) active suicidal ideation with intent/plan; (4) moderate-to-severe traumatic brain injury; (5) significant or unstable medical disturbance not controlled by medication; and (6) fMRI contraindications (e.g., metal in body, pregnancy). The authors of this study were unblinded to group membership after data collection was completed. Additionally, this is the first study from our laboratory using T1000 data to investigate cannabis use and mood/anxiety disorders.

### MID task

Neural activation to monetary wins and losses was measured using the MID task^15^. On each trial, a cue indicated potential win (circle), loss (square), or no win/loss (“neutral”, circle or square). The magnitude of reinforcement was manipulated by the location of a horizontal line on the cue. A line at the bottom of the cue represented no win or loss (neutral), a line in the middle of the cue represented a low-win or low-loss, and a line at the top of the cue represented the high-win or high-loss. Following a varied delay period, participants were required to respond to a target stimulus (white square) within a response time window to successfully obtain (positive reinforcement) or avoid (negative reinforcement) points for which they were paid for at the end of the scan. Participants on average earned $30. Task difficulty was calibrated using the reaction time (RT) measured during a practice session, so that each participant should succeed on 66% of trials. The task was divided into two runs yielding a total of 90 trials (scan time: 18 min, 44 s).

Images were acquired with a GE MRI 750 3T scanner at the Laureate Institute for Brain Research. The MID task scanning consisted of 281 contiguous echo-planar imaging volumes using the following parameters: TR/TE = 2000/27 ms, FOV/slice = 240/2.9 mm, 128 × 128 matrix, and 39 axial slices. High-resolution structural images were acquired through a 3D axial T1- weighted magnetization-prepared rapid acquisition with gradient-echo (MPRAGE) sequence, using the following parameters: TR/TE = 5/2.012 ms, FOV/slice = 240 × 192/0.9 mm, and 186 axial slices.

### Neuroimaging data analysis

Neuroimaging preprocessing was performed using the AFNI software package^37^ and included despiking, slice timing correction, coregistration to anatomical volumes, motion correction, smoothing (4 mm^3^ full width at half maximum), and normalization to the standard Montreal Neurological Institute space (MNI template, resampling voxel size was 2 mm^3^). Head displacements along the standard six directions and the associated Euclidean norm (analogous to framewise displacement) were estimated using the starting volume as the reference frame. The overall average Euclidean norm was taken as a single head motion summary statistic for each participant. Maximum head motion in any direction was also used to characterize the samples.

A two-level general linear model was used to analyze the functional data. For the first level, boxcar regressors were defined for each subject and for each epoch of the time course. The regressors modeled the BOLD response to the anticipation epoch (4 s) for six conditions: high-loss, low-loss, no-loss, no-win, low-win, and high-win (15 trials per condition). Whole-brain contrasts associated with anticipation of loss ([1 1 -2 0 0 0]) and win ([0 0 0 -2 1 1]) were calculated for second-level analyses.

Average activations were extracted from whole-brain images for voxels contained within left and right nucleus accumbens, dorsal caudate, and dorsolateral putamen, as defined from the “brainnetome” atlas^38^ (ROI indices #223-224,227-230). Data were included as dependent variables in each multivariate linear mixed effects (LME) model with hemisphere included as a fixed factor. Bilateral data were averaged across hemispheres for post-hoc correlations and visualization purposes.

### Selected participants

From the first 500 individuals from our T1000 sample, there were 370 individuals with any lifetime mood/anxiety disorder diagnoses. Those with problematic cannabis use were identified as having a lifetime diagnosis of cannabis dependence (DSM-IV) from the MINI or endorsing cannabis use at least 50 times in the past year (measured via the Customary Drinking and Drug Use Record^39^), equating to approximately weekly cannabis use. Using this criteria, 42 individuals with mood/anxiety disorders and cannabis use (“Mood/Anxiety+CB”) were identified, with 328 participants remaining for group matching.

**Fig. 1:**
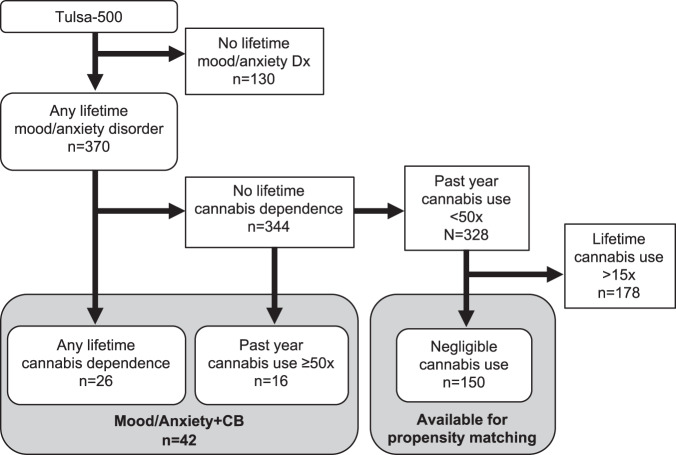
Identification criteria for psychiatric samples. All participants drawn from the first 500 of the T1000 study^35^. Participants were initially selected for having any lifetime mood and anxiety disorder diagnosis. The participants who also had a lifetime cannabis dependence diagnosis and excessive past year cannabis use (≥50 uses) comprised the Mood/Anxiety+CB group. The participants with a history of any lifetime mood and anxiety disorder diagnosis and very low lifetime cannabis use (<15 uses) comprised the eligible participants to be sampled from during propensity score-matching.

Prior to performing propensity score-matching, participants with modest levels of cannabis use were excluded as to not contaminate the comparison group with low levels of cannabis exposure. As it was not possible to match on nicotine dependence after excluding participants with any lifetime cannabis use, the acceptable lifetime use threshold for the two noncannabis groups was set at 15 uses. Hence, 178 participants were excluded, leaving 150 available for propensity score-matching. See Fig. 1 for group identification rules. Lastly, participants with poor MID task image quality or excessive head motion (mean Euclidean norm ≥0.3 mm) were excluded prior to group matching. Using this criterion, 1 participant from the Mood/Anxiety+CB group and 15 participants from the psychiatric comparison sample were excluded. Lastly, a sample of healthy controls were selected on the basis of (1) having no lifetime mood/anxiety diagnoses, (2) having no more than 15 lifetime cannabis uses, and (3) passing image quality control. A sample of 35 healthy controls were identified using these criteria. As the sample sizes here are approximately double the size of two previous fMRI studies that examined striatal activations in cannabis users^22,25^, we expected to have sufficient power to detect between-group differences.

### Propensity matching

The psychiatric comparison group was identified using a propensity score-matching approach via the “MatchIt” library in R (https://cran.r-project.org). Variables used for matching included age, sex, body mass index (BMI), head motion, and the Patient-Reported Outcomes Measurement Information System (PROMIS)^40^ current alcohol use, nicotine dependence, anxiety, and depression levels. Using the default “nearest neighbor” approach, the algorithm first used logistic regression to estimate the predicted probability of group membership status (Mood/Anxiety+CB vs. Mood/Anxiety-CB) given this set of matching covariates. Then, 1-to-1 matching was implemented to select the participant from the comparison group with the nearest predicted probability (propensity score) for each participant from the Mood/Anxiety+CB group. Hence, 41 participants with similar levels of current anxiety and depression (plus other covariates) and very low levels of cannabis use (“Mood/Anxiety-CB”) were identified. See Table 1 for comparison of group characteristics, and Table 2 for detailed psychiatric diagnoses, frequency, and presence of depressive episodes, depression severity, and medication information.

**Table 1:** Group characteristics.

Feature	Group						
	Mood/Anxiety+CB (*n* = 41)		Mood/Anxiety-CB (*n* = 41)		Healthy (*n* = 35)		
	*M*	*SD*	*M*	*SD*	*M*	*SD*	*p*
Age	29.2	7.4	30.0	10.1	31.4	11.2	0.61
Gender (Male, Female)	21, 20		21, 20		13, 22		0.52
BMI	26.0	4.0	26.7	5.1	27.5	5.5	0.40
MID head motion							
Mean euclidean norm	0.10	0.05	0.10	0.05	0.08	0.03	0.09
Max. displacement	3.08	1.2	2.58	1.1	2.48	1.1	0.04
PROMIS							
Alcohol use	49.9	4.7	48.9	5.7	45.9	5.9	0.006
Nicotine dependence	39.7	15.6	33.2	13.5	25.1	7.1	<0.001
Current anxiety	59.9	6.7	61.0	7.4	44.1	7.6	<0.001
Current depression	55.8	7.8	58.0	7.8	42.9	6.0	<0.001
CDDR							
Lifetime alcohol use	1337.3	2378.7	913.8	2969.7	299.4	860.3	0.004
Lifetime cigarette use	268272.5	1493191.1	7722.8	21725.5	663.6	2190.1	<0.001
Lifetime cannabis	14243.6	40843.5	2.8	3.7	2.9	4.3	<0.001
Past year cannabis	486.3	1546.9	0.6	1.3	0.5	1.4	<0.001
Past year stimulants	418.3	1734.4	594.3	2425.5	0.5	2.7	<0.001
Past year opioids	258.0	905.2	7.0	42.1	0.4	2.4	<0.001

Propensity score matching procedure used age, gender, BMI, Euclidean norm, and all tabulated PROMIS measures to identify Mood/Anxiety-CB group. Healthy comparison group was identified for having ≤15 lifetime cannabis uses, and no lifetime psychiatric diagnoses. *P* values from one-way ANOVAs and chi-square test (for gender) to confirm successful matching. Kruskal–Wallis tests used for CDDR measures due to lack of normality. Although greater max. head motion was demonstrated in the Mood/Anxiety+CB group relative to the healthy controls, Tukey’s post-hoc test determined this was a marginally significant difference (*p*_corr_ = 0.053). Subsequent analyses used Euclidean Norm as a covariate to account for headmotion across the task scanning session.

*BMI* body mass index, *MID* monetary incentive delay task, head motion estimates in millimeters, *PROMIS* patient-reported outcomes measurement information system^40^, *CDDR* customary drinking and drug use record^39^.

**Table 2:** Health characteristics.

Feature	Psychiatric groups				
	Mood/Anxiety-CB (*n* = 41)		Mood/Anxiety+CB(*n* = 41)		
	*N*	%	*N*	*%*	*p*
Lifetime mood/anxiety diagnoses					
Any MDD and anxiety disorders^a^	19	0.46	27	0.66	0.119
Any MDD without anxiety disorders	14	0.27	11	0.27	0.631
Any anxiety disorders without MDD	8	0.20	3	0.07	0.195
MDE frequency					
Single episode	9	0.22	7	0.17	0.781
Recurrent episode	24	0.56	31	0.76	0.159
MDE status					
Current episode	12	0.25	21	0.51	0.072
Partial remission	13	0.32	14	0.34	0.814
Full remission	8	0.20	3	0.07	0.194
Hamilton depression rating scale	*M* = 9.9	*SD* = 6.2	*M* = 11.7	*SD* = 4.7	0.24
Any lifetime DSM diagnosis					
GAD	10	0.24	19	0.46	0.065
MDD	33	0.81	38	0.83	0.195
PTSD	10	0.24	4	0.10	0.142
Panic disorder	7	0.17	7	0.17	1
Social phobia	10	0.24	8	0.20	0.790
Eating disorder	3	0.07	3	0.07	1
Lifetime substance use disorders					
Nicotine dependence	1	0.02	1	0.02	1
Alcohol dependence	12	0.29	6	0.16	0.182
Opioid dependence	12	0.29	1	0.02	0.002
Amphetamine dependence	14	0.34	3	0.07	0.006
Cocaine dependence	6	0.15	0	0	0.034
Sedative dependence	8	0.20	1	0.02	0.034
Hallucinogen dependence	1	0.02	0	0	0.314
Medication status	26	0.63	24	0.56	0.821
Class of medication					
SSRI	11	0.27	15	0.37	0.476
SNRI	4	0.10	5	0.12	0.723
Atypical antidepressant	7	0.14	8	0.20	0.775
Benzodiazepines	2	0.05	8	0.20	0.092
Opioids	6	0.15	1	0.02	0.114
Other anxiolytic	12	0.29	4	0.10	0.051
Other muscle relaxants	3	0.07	4	0.10	0.693
Stimulants	0	0	1	0.02	0.314
Mood stabilizers	2	0.05	1	0.02	0.983
Atypical antipsychotics	6	0.15	0	0	0.034
Clinical referral status	29	0.71	13	0.32	<0.01

Incidence of DSM diagnoses for relevant psychiatric disorders. All diagnoses derived from the MINI International Neuropsychiatric Interview. The first three rows summate to the sample sizes for each group, and outline the quantity of participants with a lifetime mood-and-anxiety diagnosis, mood-only, or anxiety-only diagnosis. The subsequent rows outlining MDE Frequency and Status summate to the number of participants per group with any lifetime MDD diagnosis. Rows for Any Lifetime DSM or Substance Use Disorder Diagnosis are non-cumulative per group. Clinical referral signifies recruitment from a treatment facility or health care provider versus self-referral (e.g., study advertisement). *P* values from chi-square tests, except for Hamilton Scale comparison using a 2-sample *t* test.

*MDD* major depressive disorder, *MDE m*ajor depressive episode, *GAD* generalized anxiety disorder, *PTSD* post-traumatic stress disorder, *SSRI* selective serotonin reuptake inhibitor, *SNRI s*elective norepinephrine reuptake inhibitor.

^a^Anxiety disorders included any incidence of GAD, PTSD, panic disorder, or social phobia.

### Group analysis

Nucleus accumbens, dorsal caudate, and dorsolateral putamen ROIs were the dependent variables used to test our hypothesis. First, to evaluate group differences in win-anticipation, these data were submitted to a multivariate group (Mood/Anxiety+CB, Mood/Anxiety-CB, healthy controls) by condition (high-win, low-win, neutral) LME model with a test for the interaction between the two factors using the “lme4” package in R. Group, condition, hemisphere and nuisance covariates (age, sex, BMI, and head motion) were modeled as fixed effects. Participant ID was modeled as a random effect. Specifically, the model estimated was: Activations = Group*Condition + hemisphere + age + sex + BMI + head motion + ID (random). An analogous LME model was also estimated for loss-anticipation (high-loss, low-loss, neutral). As the healthy controls, by nature of their group membership, differed from the other two groups on anxiety and depression levels, as well as alcohol use and nicotine dependence, it was not possible to covary for these measures as they were colinear with the group factor. See Supplemental Materials for model summaries (including main effects of anxiety and depression factors) estimated using the two psychiatric groups only.

In these models, the main effect of group reflected the extent to which the groups differ across condition (incentive saliency levels) while the interaction term reflects the difference in slopes across incentive saliencies (e.g., high-win, low-win, neutral) by group. To help interpret group main effects, Cohen’s *d* effect sizes were calculated for each pairwise difference in group means for each ROI by condition (e.g., Mood/Anxiety+CB vs. Healthy Controls for accumbens win-anticipations). Percent BOLD signal change for each win and loss condition were averaged across hemisphere and plotted separately by group to visualize main effects of group, condition, and their interaction. MID task behavioral performance data were analyzed using similar LME models for percent hit rate and mean RT. All estimated models were inspected for regression assumptions using the “easystats” library in R. All reported *p* values (two-sided) for model parameter estimates were corrected for three tests (three ROIs per win- or loss-anticipation) using the Holm–Bonferroni method.

### Post-hoc correlations

Significant LME main effects of group and group-by-condition interactions motivated a set of *post-hoc* tests examining associations between striatal activations and past year cannabis use. Whole-brain contrast images during win-anticipation (e.g., high-win + low-win vs. neutral) and loss-anticipation (high-loss + low-loss vs. neutral) were used to extract the average BOLD signal-change for each ROI, which were then averaged across hemisphere. These summary data were then submitted to bivariate correlations with the log10-transform of past year cannabis use. Bivariate correlations were also estimated for the log10-transform of past year opioid and stimulant use, as there were more individuals in the Mood/Anxiety+CB group with a history of opioid and stimulant use disorders (Table 2). For each drug, the Holm–Bonferroni method was used to correct for three tests across the three striatal ROIs. Drug use correlations were estimated within the Mood/Anxiety+CB group only. Lastly, an exploratory correlation matrix was generated to examine relationships between each striatal ROI, task condition, and past year drug use for the Mood/Anxiety+CB group.

## Results

### Participants

Propensity score-matching identified a comparison group (“Mood/Anxiety-CB”) of individuals equated on current depression and anxiety levels, alcohol use, and nicotine dependence. One-way ANOVAs determined that groups did not differ on key sociodemographic variables, although the healthy controls had significantly lower depression, anxiety, and drug use levels (Table 1). Nonetheless, chi-square and *t* tests between the two psychiatric groups confirmed that propensity score-matching equated these groups on current anxiety, depression, alcohol, and nicotine dependence (*p*s > 0.05; Supplemental Table S1).

The two psychiatric groups also endorsed similar medication status (e.g., use of SSRIs, etc.; Table 2), although more Mood/Anxiety+CB participants were on atypical antipsychotics. Within this group, Pearson’s point-biserial correlations between atypical antipsychotic status and striatal activations within the Mood/Anxiety+CB group determined that this use was unrelated to striatal BOLD signal responses (all *p*s > 0.05).

Lifetime prevalence rates of internalizing disorders were also highly similar between the two psychiatric groups (Table 2) and absent (by design) from the healthy control group. Any lifetime major depressive disorder (MDD) was the most common diagnosis reflected by 81% of the Mood/Anxiety+CB group and 83% of the Mood/Anxiety-CB group, followed by generalized anxiety disorder reflected by 24% and 46% of the two respective groups (*p*s > 0.05).

Although it was not possible to identify comparison groups of never-cannabis users, nearly half of the Mood/Anxiety-CB group reported no lifetime cannabis use (*n* = 20, 49%), with the remaining having extremely low levels of lifetime use (median 4 uses, maximum 12 uses). The majority of the Mood/Anxiety-CB group also reported no past year cannabis use (*n* = 30, 73%; maximum 5 uses). See Supplemental Fig. S1 and S2 for histograms of exact cannabis use levels in the Mood/Anxiety+CB group. While the Mood/Anxiety+CB group was more likely to have a history of other substance use disorders than Mood/Anxiety-CB, post-hoc tests were used to determine if any significant findings were related to past year opioid or stimulant use (see below).

Lastly, the Mood/Anxiety+CB group contained participants both with and without a lifetime history of cannabis dependence (DSM-IV). In light of concerns that these two sub-groups might reflect different neurobiological characteristics, we directly tested for striatal activation differences between these sub-groups using two-sample *t* tests. Results confirmed that the 25 participants with lifetime cannabis dependence did not differ from the 15 without a lifetime diagnosis on any of the striatal ROIs by condition. See Supplemental Table S2 for *t* test statistics.

### MID task differences

LME models identified a significant main effect of condition during win-anticipation for the nucleus accumbens (β = 1.2 × 10^−3^*, F*_1,815_ = 220.9, *p*_*corr*_ < 0.001), dorsal caudate (*β* = 1.4 × 10^−3^*, F*_1,815_ = 118.2, *p*_*corr*_ < 0.001), and dorsolateral putamen (*β* = 6.6 × 10^−4^*, F*_1,815_ = 84.7, *p*_*corr*_ < 0.001). Likewise for loss-anticipation, a significant main effect of condition was observed for the nucleus accumbens (*β* = 6.1 × 10^−4^*, F*_1,815_ = 57.7, *p*_*corr*_ < 0.001), dorsal caudate (*β* = 9.8 × 10^−4^*, F*_1,815_ = 97.7, *p* < 0.001), and dorsolateral putamen (β = 4.9 × 10^−4^*, F*_1,815_ = 48.7, *p*_*corr*_ < 0.001). As the neutral condition was modeled as the reference, the positive betas indicated a clear increase in striatal activation for each increasing win- and loss-incentive magnitudes (low, high) across groups.

There was also a significant main effect of group during win-anticipation for the nucleus accumbens (*β* = −1.3 × 10^−3^*, F*_2,120_ = 5.1, *p*_*corr*_ < 0.05), dorsal caudate (*β* = −1.3 × 10^−3^*, F*_2,120_ = 3.9, *p*_*corr*_ < 0.05), and dorsolateral putamen (*β* = −1.3 × 10^−3^*, F*_2,120_ = 5.0, *p*_*corr*_ < 0.05). Similar main effects of group during loss-anticipation were also identified for the nucleus accumbens (*β* = −1.2 × 10^−3^*, F*_2,119_ = 4.5, *p*_*corr*_ < 0.05), dorsal caudate (*β* = −1.3 × 10^−3^*, F*_2,118_ = 3.5, *p*_*corr*_ < 0.05), and dorsolateral putamen (*β* = −1.2 × 10^−3^*, F*_2,115_ = 4.5, *p*_*corr*_ < 0.05). As the healthy control group was modeled as the reference, the negative betas indicated lower striatal activation in the Mood/Anxiety+CB group relative to the healthy group across incentive magnitude. Cohen’s *d* characterized each pairwise group difference as reflective of a medium effect size (see Supplemental Table S3). Notably, the two greatest effect sizes were identified for the nucleus accumbens group differences (Mood/Anxiety+CB vs. healthy controls; win-anticipation *d* = 0.48; loss-anticipation *d* = 0.56).

**Fig. 2:**
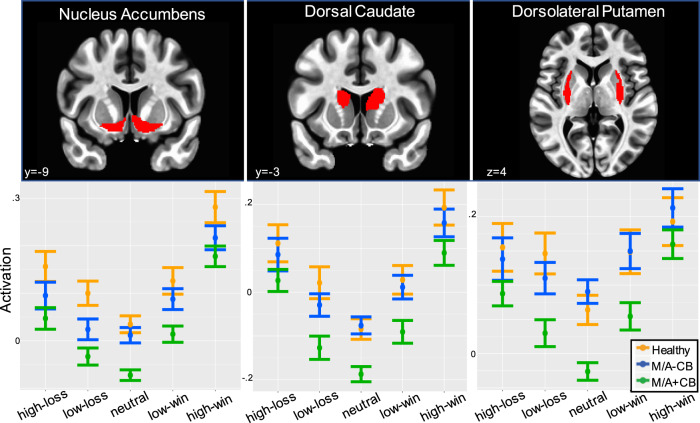
Regions of interest and activation patterns during win and loss anticipation. Mean activation (%BOLD signal change) for each condition of the monetary incentive delay task plotted by group for each striatal region of interest. Significant main effects of group were observed for both the loss (high-loss, low-loss, neutral) and reward (high-win, low-win, neutral) conditions within the nucleus accumbens (**a**), dorsal caudate (**b**), and dorsolateral putamen (**c**). For display purposes, activations were averaged across hemisphere. Error bars reflect± one standard error of the mean. Green markers represent the Mood/Anxiety+CB group (“M/A+CB”; *n* = 41), blue markers represent the matched Mood/Anxiety-CB group (“M/A-CB”; *n* = 41), and orange markers represent the healthy comparison sample (*n* = 35). All three ROIs were selected from the “brainnetome” atlas^38^.

Significant group-by-condition interactions were observed only for the dorsolateral putamen during win-anticipation (*β* = −2.5 × 10^−4^, *F*_2,815_ = 6.0, *p*_*corr*_ < 0.05) and loss-anticipation (*β* = −2.6 × 10^−4^*, F*_2,815_ = 7.2, *p*_*corr*_ < 0.05). These interaction terms indicated the slope across the magnitude of win or loss incentives (high, low, and neutral) differed by group status for this ROI. In line with the negative betas, Fig. 2 depicts an overall attenuation of activations to each win and loss condition for both the Mood/Anxiety+CB and Mood/Anxiety-CB groups relative to the healthy group within each region.

Similar LME analysis on the MID behavioral data revealed a main effect of condition during win-anticipation (*F* = 55.6, *p* < 0.001) and loss-anticipation (F = 46.3, *p* < 0.001) for RT (Supplemental Fig S3). No significant main effects of group, nor interactions were found for RT or percent hit rate data, indicating that groups performed the task similarly. See Supplementary Tables S4–S8 for model summaries and uncorrected *p* values, as well as Supplementary Tables S9–S13 for consistent model summaries using the two psychiatric samples only.

### Post-hoc correlations

**Fig. 3:**
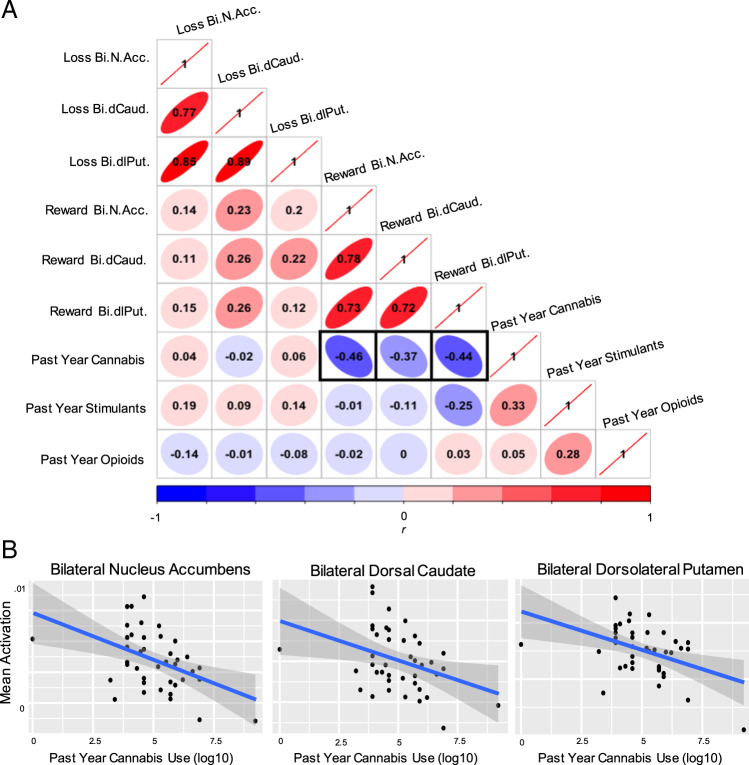
Summary correlation matrix for mood/anxiety+CB group only. Panel **a**: Correlations between all extracted striatal data and log10-transformed past year cannabis, stimulant, and opioid use within the Mood/Anxiety+CB group only. Cells depict Pearson’s *r* for each combination. Striatal activations derived from the whole-brain contrast images estimated for all loss and win conditions. Left and right ROIs were averaged across hemisphere. Bi.N.Acc = Bilateral nucleus accumbens. Bi.dCaud. = Bilateral dorsal caudate. Bi.dlPut. = Bilateral dorsolateral putamen. The three bolded cells showing the correlation between past year cannabis use and reward activations were further visualized using scatter plots shown in panel **b** (all three *p*_corr_ < 0.05).

As the Mood/Anxiety+CB group exhibited the lowest levels of reward processing, post-hoc correlations evaluated whether data exhibited a dose-response relationship with recent (past year) cannabis use; Pearson’s correlation indicated that heavier past year cannabis use was related to lower win-anticipation within bilateral nucleus accumbens (*r* = −0.46, *p*_corr_ = 0.007), dorsal caudate (*r* = −0.37, *p*_corr_ = 0.018), and dorsolateral putamen (*r* = −0.44, *p*_corr_ = 0.008) (Fig. 3a, b). Critically, the correlation matrix showed these significant dose-responses were specific to past year cannabis use and the win condition only (Fig. 3a). In addition, no dose-response was observed for past year stimulant or opioid use with any of the striatal ROIs.

## Discussion

This study tested the hypothesis that those with internalizing disorders and cannabis use would exhibit lower reward-related striatal BOLD signals than healthy controls and individuals with internalizing disorders alone. There were two main results. First, relative to both comparison groups, the Mood/Anxiety+CB group demonstrated the lowest activations in the nucleus accumbens, dorsal caudate, and dorsolateral putamen as a function of win- and loss-magnitudes. Second, individuals with heavier recent cannabis use showed greater attenuation of reward-related activation. The propensity matching and subsequent analyses showed that these effects were unlikely to be attributed to age, sex, BMI, head motion, severity of current alcohol or nicotine use, severity of anxiety or depression symptoms, or other past-year illicit drug use. Taken together, these results support the hypothesis that cannabis use interferes with processing positive and negative valenced information by reducing striatal sensitivity to reward magnitude.

The overall U-shaped striatal activation patterns were consistent with previous studies employing fMRI to track varied magnitudes of reinforcement^41^. Evidently, the Mood/Anxiety+CB group exhibited the lowest activations to the neutral condition, reflecting very low baseline levels of striatal activations. Furthermore, across all three regions, low-win activations for the Mood/Anxiety+CB group were generally equal to neutral activations for the Mood/Anxiety-CB and healthy control group. As all groups had roughly similar activations to the high-win and high-loss conditions, intermediate rewards may not be salient enough to the cannabis group to recruit a comparable level of processing resources.

The results here support the role of the striatum, and in particular, the nucleus accumbens, in tracking both the positive and negative incentive saliences of reward cues^42^, while also being sensitive to differences by cannabis use status. This sensitivity is underscored by our finding that the two greatest effect sizes were identified for win- (*d* = 0.48) and loss-anticipation (*d* = 0.56) in the nucleus accumbens. The post-hoc dose-response relationships, however, converged on the processing of win-anticipation, as the level of cannabis use was unrelated to loss activations for all three regions tested. In light of the significant main effects observed for striatal activation to losses, this lack of a dose-response with past year cannabis use suggests that hypoactivations to losses is a trait-like characteristic of the Mood/Anxiety+CB group. Nonetheless, longitudinal studies are needed to better inform this hypothesis.

Previous studies on cannabis use and depression independently reported blunted striatal activation to rewards^18,43^. As the two psychiatric groups here were equated on current depressive symptoms, the addition of cannabis use in these populations evidently magnified the severity of attenuated striatal reward activations. Hence, this study underscores the potential for cannabis to exacerbate a deficit in striatal reward activity. These findings also suggest cannabis use might have lasting detrimental effects on reward circuitry as all participants provided a negative urine drug screen prior to scanning. Furthermore, as this study analyzed mostly participants with MDD and generalized anxiety disorder, these findings are in line with the MDD literature^44^. While the adult literature using the MID task to characterize generalized anxiety is still nascent, the findings reported here are opposed to pediatric anxiety studies, which motivates the hypothesis that cannabis use precipitates a depression-like phenotype in anxious individuals. However, more studies on adult anxiety, and longitudinal studies examining cannabis use in anxious populations, are needed to understand this phenotype.

As previous studies reported that many individuals target anxious and depressive symptoms with recreational/medicinal cannabis^2,11^, we offer cautionary evidence against these trends as cannabis use correlated with neurobiological deficits in striatal reward processing. Our findings might also partially explain previous studies that reported low treatment success in anxious and depressed cannabis users^9,10^ as cannabis-related dampening of striatal reward processing might have interfered with symptom resolution. Indeed, a small longitudinal treatment study of adolescents with anxiety and depression disorders found that higher baseline striatal activations to reward anticipation predicted lower anxiety symptoms and faster recovery following cognitive behavioral therapy^33^. In addition, lower striatal functioning predicted poor treatment outcomes in depression^32^ and cannabis use disorder^34^. As long term treatment response is generally poor in anxious and depressed individuals^45^, cannabis use should therefore be discouraged by healthcare providers as it is likely a barrier to treatment success by disrupting motivational processes in the brain.

This study was limited by the cross-sectional design, which makes it impossible to determine if blunted reward activations preceded or was a consequence of cannabis or other drug use. The observed hypoactivations to rewards might have been a pre-existing risk factor which increased risk for cannabis use in these individuals as explained by the reward deficiency model of addiction^46^. However, the identified dose-response relationship with past year cannabis use informs the hypothesis that cannabis reduces striatal activations to rewards. Martz et al. provided evidence in support of this hypothesis as they demonstrated using cross-lagged models that cannabis use predicted attenuated nucleus accumbens activations two and four years later^21^. Similar findings have been reported using positron emission tomography of striatal dopamine release in participants with cannabis dependence relative to controls^47^. Therefore, cannabis use is likely to have a lasting antagonistic effect on striatal functioning.

The propensity score-matching technique used here was a very rigorous approach to roughly equate the two psychiatric groups on features that would have otherwise confounded causal interpretations. While it would still be incautious to assert a causal relationship between cannabis use and the observed attenuation of reward activations, propensity matching diminished the likelihood that the striatal differences between the two psychiatric samples were due to anxiety and depression symptoms, alcohol and nicotine use, socio-demographic variables, or head motion during scanning. Although the two psychiatric samples were found to have similar depression characteristics and medication uses, there were more participants in the Mood/Anxiety+CB group referred from clinical sources, which limits the generalizability of the findings. Lastly, the samples were an admixture of various psychopathologies, and although diagnoses were largely balanced across psychiatric groups, future studies may attempt to reproduce findings for participants in singular diagnostic categories. Additional limitations of the study include the lack of data on number of hospitalizations for each disorder, age of first diagnosis for each disorder, and age of first use for each drug of abuse. This data would help substantiate (but not verify) causal interpretations. Moreover, the self-reported and relatively imprecise nature of the drug use measures (i.e., a single value assigned for last year and lifetime usage) are also considered a limitation. As the Tulsa 1000 study was not designed to study drug use *per se*, future studies on similar topics would benefit from carefully collected timeline follow back instruments, and more fine-grained drug use levels and urinalysis data collected over time.

To continue to inform causal relationships, studies are needed to examine the extent to which striatal activations recover in anxious and depressed patients following cannabis cessation. Previous studies using behavioral activation^48^ or escitalopram^49^ to treat depression have both reported a recovery of striatal activation following treatment. Therefore, it is hypothesized that treatments targeting cannabis use in these individuals might also be effective in normalizing or adjusting their reward activations to the level of noncannabis using peers. Nonetheless, incorporating treatment for cannabis use in these populations would likely be beneficial regardless of the potential impact on neurobiology^4,5,9,10^.

As this study probed subcortical reward circuitry, future studies should examine cortical reward circuitry including the ventromedial prefrontal, anterior cingulate, and insular cortices. And although the correlation matrix showed the striatal regions largely coactivated during wins or losses, network-based analyses are needed to better elucidate cannabis-related disruptions to the reward circuitry in these populations. In terms of the interrogated sample, this study examined relatively heavy cannabis use, therefore, it is unknown if modest use might have similar detrimental (or possibly therapeutic) effects on reward processing in populations with mood and anxiety disorders. Hence, future studies are needed to examine these groups of individuals with nuanced cannabis use, including a control group of cannabis users without elevated mood/anxiety symptoms. There was also a higher prevalence of other substance use disorders in the Mood/Anxiety+CB, therefore, chronicity of other drug use might have influenced these results. Lastly, participants were drawn from a convenient sample of treatment-seeking individuals. Studies designed to specifically examine mood/anxiety and cannabis use disorders in individuals without other substance use disorders are therefore needed to corroborate the findings reported here.

## Supplementary information

Supplemental Materials

## Data Availability

R scripts for propensity score matching and regression modeling are available upon request to the corresponding author.
