# Supplementary material for: Attenuated reward activations associated with cannabis use in anxious/depressed individuals
Source: Transl Psychiatry. 2020 Jun 15;10:189. doi: 10.1038/s41398-020-0807-9 (PMC7295993; doi:10.1038/s41398-020-0807-9)
Supplement: Supplementary file 1 — Supplemental Materials [file 41398_2020_807_MOESM1_ESM.pdf]

Supplemental Materials

Model coefficients were estimated using 'lme4' in R. All tabulated factors were estimated as fixed effects, while each subject was modelled as a random effect. Significant group by condition interaction effects are displayed in bold. BMI = Body mass index. Alcohol, Anxiety, Depression, and Nicotine Dependence were measured using the Patient-Reported Outcomes Measurement Information System (PROMIS). MID Head motion reflects the mean Euclidean norm across the standard six direction head motion estimates.

Table S1: Two psychiatric group characteristic comparisons

Feature	Group				
	Mood/Anxiety+CB (n=41)		Mood/Anxiety-CB (n=41)		
	M	*SD*	*M*	*SD*	*p*
Age	29.2	7.4	30.0	10.1	.69
Gender (Male, Female)	21, 20		21, 20		1.0
BMI	26.0	4.0	26.7	5.1	.50
MID Head Motion	0.10	.05	.10	.05	.79
PROMIS					
Alcohol Use	49.9	4.7	48.9	5.7	.43
Nicotine Dependence	39.7	15.6	33.2	13.5	.05
Current Anxiety	59.9	6.7	61.0	7.4	.47
Current Depression	55.8	7.8	58.0	7.8	.19
CDDR					
Lifetime Alcohol Use	1337.3	2378.7	913.8	2969.7	.49
Lifetime Cigarette Use	268272.5	1493191.1	7722.8	21725.5	.29
Lifetime Cannabis	14243.6	40843.5	2.8	3.7	<.001
Past Year Cannabis	486.3	1546.9	0.63	1.3	<.001
Past Year Stimulants	418.3	1734.4	594.3	2425.5	.71
Past Year Opioids	258.0	905.2	7.0	42.1	.08

Mood/Anxiety+CB contains participants identified for having: (1) a lifetime diagnosis any mood or anxiety disorder diagnosis; and (2) either a lifetime diagnosis of DSM-IV cannabis dependence or having used cannabis at least 50 times in the past year. Mood/Anxiety-CB contains similar participants with mood or anxiety disorder diagnoses with no more than 15 lifetime cannabis uses. All features (except CDDR measures) were used in propensity score-matching and used as nuisance covariates in the LME models estimated on these two groups only (**Tables S9 – S13**). P-values from two-group *t*-tests and a chi-square test (for gender) confirmed successful matching. Non-parametric tests were used for CDDR measures due to non-normality. BMI = Body Mass Index. MID = Monetary incentive delay task. Head motion represents mean Euclidean norm (mm). PROMIS = Patient-reported outcomes measurement information system. CDDR = Customary Drinking and Drug Use Record.

Table S2: Comparison of Mood/Anxiety+CB Participants With (n=25) vs. Without a Lifetime Cannabis Use Disorder Diagnosis (n=16)

Region	Condition	*t*	*p*
Bilateral Nucleus Accumbens	Win-Anticipation	0.35	.73
	Loss-Anticipation	0.25	.80
Bilateral Dorsal Caudate	Win-Anticipation	0.94	.35
	Loss-Anticipation	0.10	.92
Bilateral Dorsolateral Putamen	Win-Anticipation	1.1	.29
	Loss-Anticipation	0.67	.51

Results from two-sample t-tests using the average activations across hemispheres for three striatal regions. T-test indicated the two groups of participants were

Table S3: Cohen's *d* for Pairwise Comparisons for Mood/Anxiety+CB vs. Other Groups

Region	Condition	*Contrast*	*Cohen's d*
Bilateral Nucleus Accumbens	*Win-Anticipation*	Mood/Anxiety+CB vs. Healthy Controls	0.48
		Mood/Anxiety+CB vs. Mood/Anxiety-CB	0.34
	Loss-Anticipation	Mood/Anxiety+CB vs. Healthy Controls	0.56
		Mood/Anxiety+CB vs. Mood/Anxiety-CB	0.35
Bilateral Dorsal Caudate	*Win-Anticipation*	Mood/Anxiety+CB vs. Healthy Controls	0.37
		Mood/Anxiety+CB vs. Mood/Anxiety-CB	0.37
	Loss-Anticipation	Mood/Anxiety+CB vs. Healthy Controls	0.39
		Mood/Anxiety+CB vs. Mood/Anxiety-CB	0.36
Bilateral Dorsolateral Putamen	*Win-Anticipation*	Mood/Anxiety+CB vs. Healthy Controls	0.33
		Mood/Anxiety+CB vs. Mood/Anxiety-CB	0.44
	Loss-Anticipation	Mood/Anxiety+CB vs. Healthy Controls	0.41
		Mood/Anxiety+CB vs. Mood/Anxiety-CB	0.44

Table S4: Estimated model coefficients for nucleus accumbens

Condition	Factor	Beta	*Standard Error*	*p*
Win Anticipation	Group x Condition	1.24e-05	1.10e-04	.91
	Group	-1.25e-03	4.16e-04	<.01
	Condition	1.20e-03	8.15e+02	<.01
	Age	-3.88e-05	1.81e-05	.03
	Sex	-4.17e-04	3.223e-04	.10
	BMI	-2.67e-05	3.57e-05	.46
	Hemisphere	6.29e-05	7.35e-05	.39
	MID Head Motion	4.75e-03	3.83e-03	.22
Loss Anticipation	Group x Condition	3.31e-05	1.10e-04	.76
	Group	1.22e-03	4.27e-04	<.01
	Condition	-6.12e-04	8.06e-05	<.01
	Age	-3.05e-05	1.86e-05	.10
	Sex	-3.77e-04	3.31e-04	.26
	BMI	-1.26e-05	3.67e-05	.73
	Hemisphere	7.76e-05	7.31e-05	.29
	MID Head Motion	3.48e-03	3.94e-03	.38

Table S5: Estimated model coefficients for dorsal caudate

Condition	Factor	Beta	*Standard Error*	*p*
Win Anticipation	Group x Condition	-1.56e-05	1.35e-04	.91
	Group	-1.34e-03	5.47e-04	.02
	Condition	1.36e-03	9.93e-05	<.01
	Age	5.77e-06	2.40e-05	.60
	Sex	-4.191e-04	4.25e-04	.33
	BMI	-5.31e-05	4.71e-05	.26
	Hemisphere	2.19e-04	9.01e-05	.02
	MID Head Motion	1.29e-02	5.05e-03	.01
Loss Anticipation	Group x Condition	8.75e-04	3.40e-03	.80
	Group	2.3e-02	1.16e-02	.02
	Condition	1.71e-02	2.52e-03	<.01
	Age	1.98e-04	5.00e-05	.69
	Sex	-1.24e-02	9.13e-03	.18
	BMI	4.94e-05	1.01e-03	.96
	Hemisphere	1.95e-04	9.03e-05	.03
	MID Head Motion	1.11e-02	5.3e-03	.04

Table S6: Estimated model coefficients for dorsolateral putamen

Condition	Factor	Beta	*Standard Error*	*p*
Win Anticipation	Group x Condition	2.55e-04	9.81e-05	<.01
	Group	-1.277e-03	4.91e-04	.01
	Condition	6.63e-04	7.21e-05	<.01
	Age	-1.62e-05	2.16e-05	.46
	Sex	1.25e-04	3.84e-04	.75
	BMI	-9.15e-05	4.26e-05	.03
	Hemisphere	-8.70e-05	6.54e-05	.18
	MID Head Motion	6.17e-03	4.56e-03	.18
Loss Anticipation	Group x Condition	-7.75e-05	9.55e-05	.42
	Group	-1.19e-03	4.94e-04	.02
	Condition	4.90e-04	7.02e-05	<.01
	Age	-9.30e-06	2.17e-05	.67
	Sex	1.13e-04	3.87e-04	.77
	BMI	-7.65e-05	4.29e-05	.08
	Hemisphere	-8.74e-05	6.36e-05	.17
	MID Head Motion	3.64e-03	4.60e-03	.43

Table S7: Estimated model coefficients for MID task mean reaction time

Condition	Factor	Beta	*Standard Error*	*p*
Win Anticipation	Group x Condition	3.11e-03	3.25e-03	.34
	Group	2.31e-02	1.15e-02	.05
	Condition	-1.71e-02	2.40e-03	<.01
	Age	2.62e-04	4.97e-04	.60
	Sex	-1.52e-02	9.08e-03	.10
	BMI	3.26e-04	1.00e-03	.75
Loss Anticipation	Group x Condition	8.75e-04	3.40e-03	.80
	Group	2.3e-02	1.16e-02	.05
	Condition	1.71e-02	2.52e-03	<.01
	Age	1.98e-04	5.00e-05	.69
	Sex	-1.24e-02	9.13e-03	.18
	BMI	4.94e-05	1.01e-03	.96

Table S8: Estimated model coefficients for MID task percent hit rate

Condition	Factor	Beta	*Standard Error*	*p*
Win Anticipation	Group x Condition	0.31	1.26	.81
	Group	2.49	2.32	.29
	Condition	0.29	0.93	.76
	Age	-1.74e-04	9.37e-02	.99
	Sex	0.22	0.17	.90
	BMI	-0.13	0.19	.50
Loss Anticipation	Group x Condition	-1.46	1.20	.22
	Group	2.68	2.32	.25
	Condition	0.14	0.88	.87
	Age	0.02	0.09	.86
	Sex	0.23	1.73	.89
	BMI	-0.10	0.19	.61

Table S9: Estimated model coefficients for nucleus accumbens: Two psychiatric groups only

Condition	Factor	Beta	Standard Error	*p*
Win Anticipation	Group x Condition	-3.750e-04	1.166e-04	.001
	Group	-1.695e-04	3.565e-04	.64
	Condition	1.574e-03	9.675e-05	<.001
	Side	1.492e-04	8.960e-05	.10
	Age	1.635e-05	2.314e-05	.48
	Sex	-1.753e-04	3.507e-04	.62
	BMI	2.057e-06	4.143e-05	.96
	Alcohol	2.358e-05	3.502e-05	.50
	Anxiety	-6.810e-05	3.283e-05	.04
	Depression	6.947e-05	3.016e-05	.02
	Nicotine Dependence	-2.832e-05	1.369e-05	.04
	MID Head Motion	2.259e-03	3.846e-03	.56
Loss Anticipation	Group x Condition	-2.342e-04	2.861e-03	.03
	Group	-2.249e-04	3.800e-04	.56
	Condition	6.931e-04	8.964e-05	<.001
	Side	9.843e-05	8.302e-05	.24
	Age	2.136e-05	2.478e-05	.40
	Sex	-4.762e-05	3.749e-04	.90
	BMI	4.566e-06	4.428e-05	.92
	Alcohol	2.364e-05	3.751e-05	.53
	Anxiety	-6.768e-05	3.509e-05	.06
	Depression	7.034e-05	3.228e-05	.03
	Nicotine Dependence	-3.142e-05	1.470e-05	.04
	MID Head Motion	1.076e-03	4.114e-03	.79

Table S10: Estimated model coefficients for dorsal caudate: Two psychiatric groups only

Condition	Factor	Beta	*Standard Error*	*p*
Win Anticipation	**Group x Condition**	**-5.008e-04**	**1.370e-04**	**<.001**
	Group	-3.251e-04	4.711e-04	.49
	Condition	1.598e-03	1.136e-04	<.001
	Side	2.283e-04	1.053e-04	.03
	Age	5.005e-05	3.071e-05	.11
	Sex	-4.984e-04	4.646e-04	.29
	BMI	-3.428e-05	5.489e-05	.53
	Alcohol	1.025e-05	4.648e-05	.83
	Anxiety	-4.711e-05	4.350e-05	.28
	Depression	7.924e-05	4.001e-05	.05
	Nicotine Dependence	-2.860e-05	1.821e-05	.12
	MID Head Motion	1.307e-02	5.099e-03	.01
Loss Anticipation	Group x Condition	-1.622e-04	1.266e-04	.20
	Group	-5.069e-04	4.908e-04	.31
	Condition	1.005e-03	1.051e-04	<.001
	Side	1.170e-04	9.730e-05	.23
	Age	6.358e-05	3.208e-05	.05
	Sex	-4.301e-04	4.849e-04	.38
	BMI	-1.705e-05	5.727e-05	.77
	Alcohol	2.484e-05	4.857e-05	.61
	Anxiety	-5.751e-05	4.539e-05	.21
	Depression	8.352e-05	4.179e-05	.05
	Nicotine Dependence	-3.244e-05	1.906e-05	.10
	MID Head Motion	1.109e-02	5.324e-03	.04

Table S11: Estimated model coefficients for dorsolateral putamen: Two psychiatric groups only

Condition	Factor	Beta	*Standard Error*	*p*
Win Anticipation	**Group x Condition**	**-2.579e-04**	**1.018e-04**	**.01**
	Group	-5.583e-04	4.321e-04	.20
	Condition	9.874e-04	8.445e-05	<.001
	Side	5.639e-05	7.821e-05	.47
	Age	4.129e-05	2.830e-05	.15
	Sex	3.446e-04	4.274e-04	.42
	BMI	-4.776e-05	5.047e-05	.35
	Alcohol	1.322e-05	4.285e-05	.76
	Anxiety	-4.747e-05	4.000e-05	.24
	Depression	6.029e-05	3.685e-05	.11
	Nicotine Dependence	-2.228e-05	1.683e-05	.19
	MID Head Motion	5.301e-03	4.694e-03	.26
Loss Anticipation	Group x Condition	-6.182e-05	9.375e-05	.51
	Group	-7.148e-04	4.337e-04	.10
	Condition	3.454e-04	7.776e-05	<.001
	Side	1.344e-05	7.202e-05	.85
	Age	5.206e-05	2.844e-05	.07
	Sex	3.262e-04	4.294e-04	.45
	BMI	-4.001e-05	5.069e-05	.43
	Alcohol	1.723e-05	4.307e-05	.69
	Anxiety	-4.863e-05	4.019e-05	.23
	Depression	6.466e-05	3.704e-05	.09
	Nicotine Dependence	-2.403e-05	1.693e-05	.16
	MID Head Motion	2.157e-03	4.717e-03	.65

Table S12: Estimated model coefficients for MID task mean reaction time: Two psychiatric groups only

Condition	Factor	Beta	*Standard Error*	*p*
Win Anticipation	Group x Condition	4.146e-03	3.533e-03	.24
	Group	3.463e-03	1.190e-02	.77
	Condition	-1.451e-02	2.933e-03	<.001
	Age	-5.128e-04	7.353e-04	.49
	Sex	-1.925e-02	1.179e-02	.11
	BMI	-8.762e-04	1.393e-03	.53
	Alcohol	2.043e-05	1.177e-03	.99
	Anxiety	2.561e-04	1.104e-03	.82
	Depression	5.914e-04	1.015e-03	.56
	Nicotine Dependence	4.724e-04	4.374e-04	.28
Loss Anticipation	Group x Condition	9.807e-04	3.590e-03	.79
	Group	3.950e-03	1.201e-02	.74
	Condition	1.160e-02	2.979e-03	<.001
	Age	-5.336e-04	7.420e-04	.47
	Sex	-1.336e-02	1.190e-02	.27
	BMI	-1.435e-03	1.405e-03	.31
	Alcohol	3.437e-04	1.188e-03	.77
	Anxiety	3.335e-06	1.114e-03	.99
	Depression	1.379e-04	1.024e-03	.89
	Nicotine Dependence	3.490e-04	4.414e-04	.43

Table S13: Estimated model coefficients for MID task percent hit rate: Two psychiatric groups only

Condition	Factor	Beta	*Standard Error*	*p*
Win Anticipation	Group x Condition	0.199	1.22573	.87
	Group	3.362	2.22247	.13
	Condition	0.892	1.01677	.38
	Age	0.027	0.13164	.84
	Sex	0.692	2.12115	.75
	BMI	-0.017	0.25043	.95
	Alcohol	-0.011	0.20769	.96
	Anxiety	0.214	0.19953	.29
	Depression	-0.240	0.18093	.19
	Nicotine Dependence	-0.022	0.07622	.78
Loss Anticipation	Group x Condition	0.390	1.21158	.75
	Group	4.377	2.22757	.05
	Condition	-2.044	1.00503	.04
	Age	0.091	0.13222	.50
	Sex	0.830	2.12992	.70
	BMI	-0.057	0.25152	.82
	Alcohol	-0.050	0.20873	.81
	Anxiety	0.067	0.20030	.74
	Depression	-0.185	0.18173	.31
	Nicotine Dependence	-0.032	0.07665	.68

Supplemental Figures

Figure S1: Histograms of Lifetime Cannabis Use

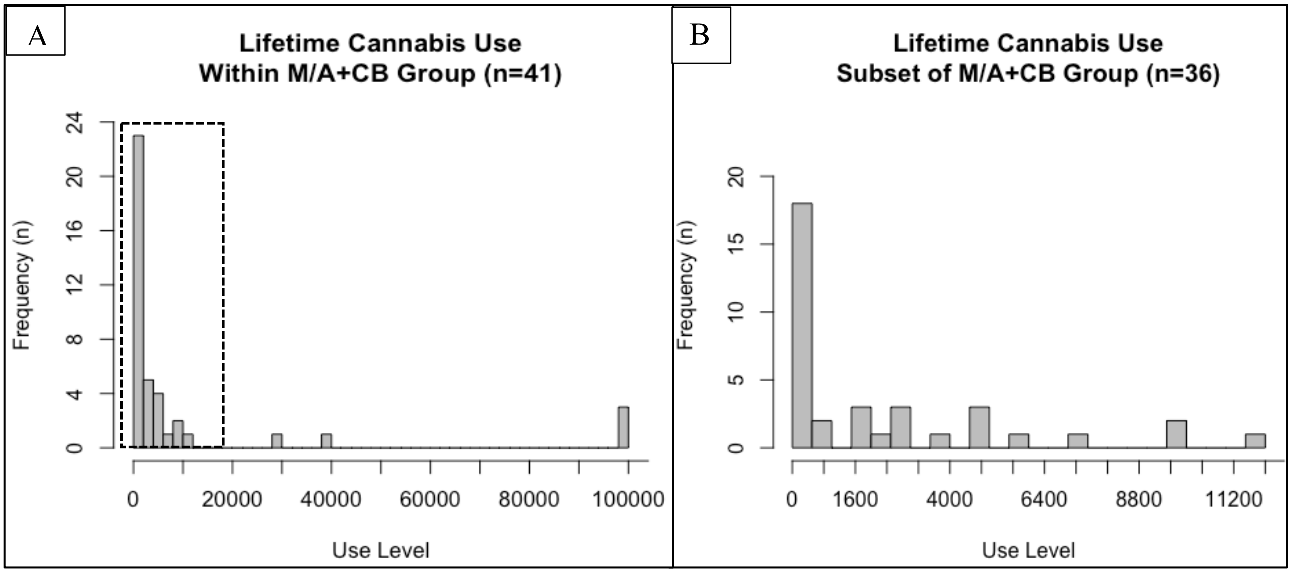

Amount of lifetime cannabis use occasions (A) across full sample of Mood/Anxiety+CB ("M/A+CB") participants and (B) the subset of participants with a lifetime use truncated at 20000 (those contained in the dashed box found in panel A).

Figure S2: Histogram of Last Year Cannabis USe

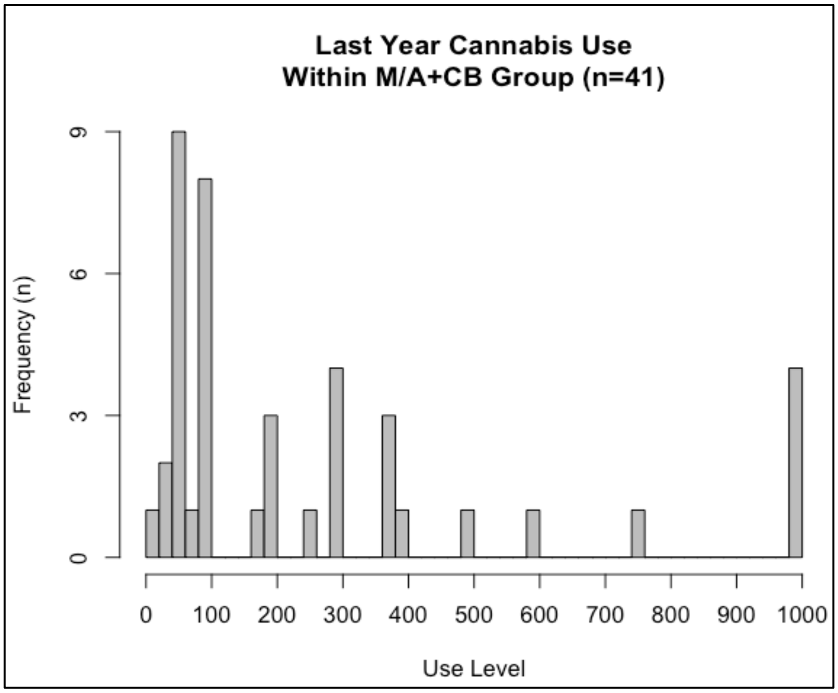

Amount of last year cannabis use across full sample of Mood/Anxiety+CB ("M/A+CB") groups.

Figure S3: MID Behavioral Task Performance

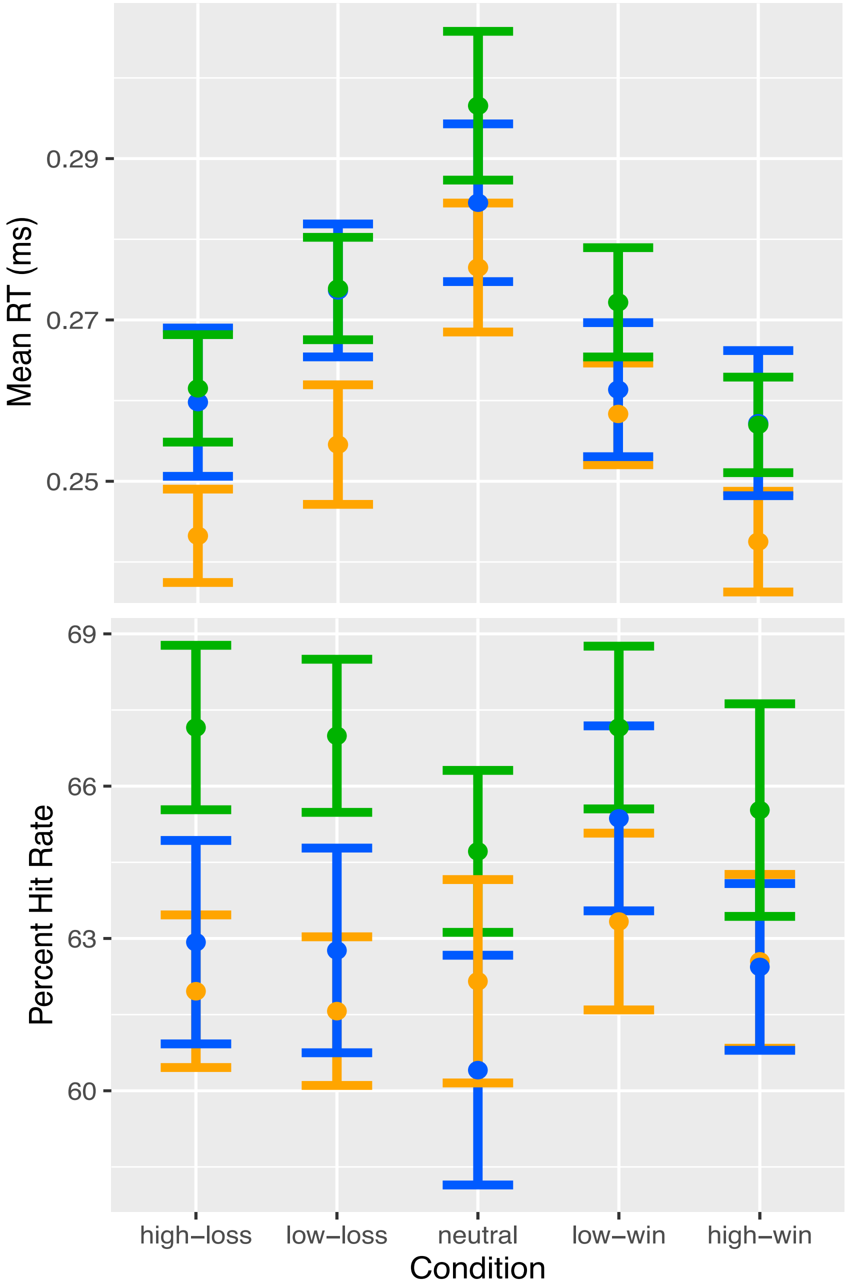

Behavioral task performance data for Mood/Anxiety+CB (green), Mood/Anxiety-CB (blue), and healthy control (yellow) groups. Top panel displays mean reaction time (RT) in milliseconds, and bottom panel displays percent hit rate (percentage of correct responses), for each condition by group. Linear mixed effects models did not identify any significant main effect of group, nor group by condition interaction, for the loss or win conditions for either mean RT or percent hit rate data.
